# Genome-wide association study of drought-related resistance traits in *Aegilops tauschii*

**DOI:** 10.1590/1678-4685-GMB-2015-0232

**Published:** 2016-07-07

**Authors:** Peng Qin, Yu Lin, Yaodong Hu, Kun Liu, Shuangshuang Mao, Zhanyi Li, Jirui Wang, Yaxi Liu, Yuming Wei, Youliang Zheng

**Affiliations:** 1 Triticeae Research Institute, Sichuan Agricultural University, Wenjiang, Chengdu, China; 2 College of Agronomy and Biotechnology, Yunnan Agricultural University, Kunming, China; 3 Institute of Animal Genetics and Breeding, College of Animal Science and Technology, Sichuan Agricultural University, Chengdu, China; 4 Farm Animal Genetic Resources Exploration and Innovation Key Laboratory of Sichuan Province, Sichuan Agricultural University, Chengdu, China

**Keywords:** Aegilops tauschii, drought resistance, genome-wide association study, single nucleotide polymorphism, wheat

## Abstract

The D-genome progenitor of wheat (*Triticum aestivum*), *Aegilops tauschii*, possesses numerous genes for resistance to abiotic stresses, including drought. Therefore, information on the genetic architecture of *A. tauschii* can aid the development of drought-resistant wheat varieties. Here, we evaluated 13 traits in 373 *A. tauschii* accessions grown under normal and polyethylene glycol-simulated drought stress conditions and performed a genome-wide association study using 7,185 single nucleotide polymorphism (SNP) markers. We identified 208 and 28 SNPs associated with all traits using the general linear model and mixed linear model, respectively, while both models detected 25 significant SNPs with genome-wide distribution. Public database searches revealed several candidate/flanking genes related to drought resistance that were grouped into three categories according to the type of encoded protein (enzyme, storage protein, and drought-induced protein). This study provided essential information for SNPs and genes related to drought resistance in *A. tauschii* and wheat, and represents a foundation for breeding drought-resistant wheat cultivars using marker-assisted selection.

## Introduction

The current global climate change is projected to have a significant impact on temperature and precipitation profiles, with consequent increases in drought incidence and severity. It is known that severe drought occurs in nearly half of the world's countries (Wilhite and Glantz, 1985). Since drought is probably the major abiotic factor limiting yields, the development of crops that are high yielding under environmentally stressful conditions is essential (Ergen and Budak, 2009; Fleury *et al.*, 2010).

Wheat (*Triticum* spp.) is the leading human food source, accounting for more than half of the world's total food consumption (Ergen and Budak, 2009; Habash *et al.*, 2009); therefore, it is a major target for the development of cultivars that are high-yielding under water-limited conditions. For drought-related research and the improvement of modern crop varieties, plants exhibiting high drought resistance are the most suitable targets and the most promising sources of drought-related genes and gene regions. Many wild species also retain superior genetic resources that have not yet been investigated. One such species is *Aegilops tauschii,* the diploid D-genome progenitor of hexaploid wheat (*T. aestivum*). *A. tauschii* is more drought resistant than *T. aestivum* and wild emmer wheat (*T. dicoccoides*) and harbors drought-resistance traits that were lost during the breeding processes (Ashraf *et al.*, 2009). Breeders have increasingly focused on *A. tauschii*, since an understanding of the genetic basis of drought resistance in *A. tauschii* can contribute to the development of drought-resistant wheat cultivars.

Drought resistance is a quantitative trait with a complex phenotype affected by plant development stages (Budak *et al.*, 2013). Linkage analysis is the most commonly used strategy for detecting quantitative trait loci (QTLs) in plants; however, linkage mapping using biparental crosses has some serious limitations. This method can only reveal information regarding two alleles at a given locus, or a few loci segregating in a studied population. In addition, the genetic resolution of detected QTLs is poor (Holland, 2007; Navakode *et al.*, 2014). Furthermore, linkage analysis can only sample a small fraction of all possible alleles in the parental source population, while the development of mapping populations is costly and time-consuming.

Association mapping (AM), also known as linkage disequilibrium mapping, relies on existing natural populations or specially designed populations to overcome the constraints of linkage mapping (Pasam *et al.*, 2012). This technique is a powerful tool to resolve complex trait variation and identify different loci and/or novel and superior alleles in natural populations (Zhu *et al.*, 2008). In recent years, association studies have been extensively used to discover and validate QTLs or genes for important traits and to map candidate genes in many crop plants, including wheat. The benefit of this method over traditional bi-parental mapping approaches depends on the extent of linkage (Huang *et al.*, 2010; Kump *et al.*, 2011; Erena *et al.*, 2013). In wheat, different association panels have been used in many AM studies to identify loci controlling agronomic (Breseghello and Sorrells, 2006; Crossa *et al.*, 2007; Neumann *et al.*, 2007; Bordes *et al.*, 2013) and quality (Ravel *et al.*, 2009; Bordes *et al.*, 2011) traits.

Only a few genome-wide association studies have been carried out in *A. tauschii* for drought resistance traits. Here, we aimed to: 1) investigate marker-trait associations for drought resistance based on a genome-wide AM approach using single nucleotide polymorphism (SNP) markers in a core collection of 373 *A. tauschii* accessions of diverse origin; 2) identify SNPs highly associated with drought resistance traits; and 3) search for candidate genes controlling these traits. This study could provide important information for cloning genes related to drought-resistance in *A. tauschii* and develop resistant wheat cultivars using marker-assisted selection.

## Material and Methods

### Plant materials and phenotypic evaluation

The natural population used for the association analysis comprised of 373 *A. tauschii* accessions collected by the Triticeae Research Institute of Sichuan Agricultural University. *A. tauschii* plants were grown in a phytotron in Wenjiang, Sichuan Province, China, from September 2012 to March 2013 and evaluated under normal conditions (NC) and polyethylene glycol (PEG)-simulated drought-stress conditions (SC) in a completely randomized design with four replications per treatment. Hydroponic tanks were filled with standard Hoagland's nutrient solution (1 mM KH_2_PO_4_, 2 mM MgSO_4_7H_2_O, 4 mM CaNO_3_4H_2_O, 6 mM KNO_3_, 0.046 mM H_3_BO_3_, 0.76 μM ZnSO_4_, 0.32 μM CuSO_4_5H_2_O, 9.146 μM MnCl_2_, 0.0161 μM (NH_4_)_6_ MoO_4_4H_2_O, and 100 μM NaFeEDTA; Hoagland and Arnon, 1950) with or without PEG (19.2%) for SC and NC, respectively. Seedlings were grown at a temperature of 25/22 ± 1 °C day/night, relative humidity of 65/85% day/night, and a 16-h photoperiod with 500 mmolm^-2^s^-1^ photon flux density at the level of plant canopy.

Uniform seedlings were transferred to the phytotron 8 d after germination and evaluated 22 d later with a WinRHizo Pro 2008a image analysis system (Régent Instruments, Quebec, Canada) for the following traits: root length (RL), root diameter (RD), the number of root tips (RT), and the number of roots with a diameter of 0.000-0.500 mm (TNOR). The plants were then separated into shoots and roots for measuring total fresh weight (TFW), root fresh weight (RFW), shoot fresh weight (SFW), and shoot height (SH). To determine total dry weight (TDW), root dry weight (RDW), and shoot dry weight (SDW), shoots and roots were stored in paper bags, heated at 105 °C for 30 min to kill the cells, and dried at 75 °C until a constant mass was obtained.

Descriptive statistics, correlation analysis, analysis of variance, principal component analysis and multiple linear stepwise regressions were conducted for all traits using IBM SPSS Statistics for Windows 20.0 (IBM Corp., Chicago, IL, USA). Heritability was calculated as follows (Smith *et al.*, 1998):

H = VG / (VG + VE),

where VG and VE represent estimates of genetic and environmental variances, respectively.

In order to eliminate individual variation resulting from inherent genetic differences unrelated to drought resistance, the drought resistance index (DI) was used as a standardizing measure across *A. tauschii* accessions and calculated as follows (Bouslama and Schapaugh, 1950):

$$\text{DI =}{\text{T}}_{\text{SC}}/{\text{T}}_{\text{NC}},$$

where T_SC_ and T_NC_ are the traits measured for each plant under SC and NC, respectively.

We also calculated the weighted comprehensive evaluation value (D value) for each genotype as follows (Xie, 1993; Zhou *et al.*, 2003):

$$\text{D =}\;\sum_{\text{j}\;\text{=}\;\text{1}}^{\text{n}}\left[{\text{u(x}}_{\text{j}}\text{)}\times {\text{W}}_{\text{j}}\right]$$

where W_j_ is the weighting variable calculated as:

$${\text{W}}_{\text{j}}=\frac{{\text{P}}_{\text{j}}}{\sum_{\text{j}\;=\;\text{a}}^{\text{n}}{\text{P}}_{\text{j}}}$$

with P_j_ being the percent of variance and u(Xj) the membership function value calculated as:

$${\text{u(x}}_{\text{j}}\text{)}\;\text{=}\;\frac{{\text{X}}_{\text{j}}-{\text{X}}_{\min}}{{\text{X}}_{\max}-{\text{X}}_{\min}}$$

### 10K Infinium iSelect SNP array and SNP genotyping

The construction of the *A. tauschii* 10K SNP array was described previously by Luo *et al.* (2014). A total of 7,185 SNP markers was mapped to an *A. tauschii* genetic map and a physical map built by bacterial artificial chromosome clones (Luo *et al.*, 2014). SNPs were assayed according to the manufacturer's protocol (Illumina, San Diego, CA, USA) at the Genome Center, University of California, Davis, CA, USA. Normalized Cy3 and Cy5 fluorescence for each DNA sample was graphed using Genome Studio (Illumina, San Diego, CA, USA), resulting in genotype clustering for each SNP marker. SNP genotyping was carried out as described previously by Wang *et al.* (2013).

### Population structure

Population structure was estimated with a set of 7,185 SNP markers mapped to the *A. tauschii* genetic map using STRUCTURE 2.3.3, which implements a model-based Bayesian cluster analysis (Pritchard *et al.*, 2000; Wang *et al.*, 2013). The linkage ancestry model and the allele frequency-correlated model were used. A total of 100 burn-in iterations followed by 100 Markov chain Monte Carlo iterations for *K* = 1 to 10 clusters were used to identify the optimal range of *K*. Five runs were performed separately for each value of *K*, and the optimal *K*-value was determined using the delta *K* method (Evanno *et al.*, 2005). Using *K* = 4 (Wang *et al.*, 2013), the population was divided into Subp1, Subp2, Subp3, Subp4, and mixed individuals.

### Genome-wide association study

Marker-trait associations were calculated in Tassel 2.1 (Bradbury *et al.*, 2007) using both the general linear model (GLM) and the mixed linear model (MLM). Both models used 6,905 SNP markers with a minor allele frequency threshold (> 0.05). To correct the population structure, the GLM incorporated a *Q*-matrix and the MLM incorporated *Q*- and *K*-matrices. The Bonferroni-corrected threshold at *α* = 1 (Yang *et al.*, 2014) was used as the cutoff value, which was 144.823 × 10^-6^ with a corresponding -log *p*-value of 3.839. Significant markers were visualized with a Manhattan plot drawn in R 3.03 (http://www.r-project.org/). Important *p*-value distributions (observed vs. cumulative *p*-values on a -log_10_ scale) were displayed in a quantile-quantile plot drawn in R. To find candidate genes, flanking genes, and trait-related proteins, we performed a Basic Local Alignment Search Tool (BLAST) search against the International Wheat Genome Sequencing Consortium database (IWGSC; http://www.wheatgenome.org/) using SNP sequences. The IWGSC BLAST results were used to perform a BLAST search of the National Center for Biotechnology Information (NCBI) database (http://www.ncbi.nlm.nih.gov/) and then a direct BLASTx search of the NCBI database.

## Results

### Phenotypic evaluation

Significant phenotypic variation was observed for all traits, and the means were significantly different between NC and SC (Table 1). The mean values of the root to shoot ratio of fresh weight (FRS), the root to shoot ratio of dry weight (DRS), RT, and RL were higher under SC, whereas RFW, SFW, RDW, SDW, SH, TFW, TDW, RD, and TNOR were lower under SC compared with those under NC (Table 1). Significant differences between NC and SC were observed for all traits, except for RFW, FRS, TFW, and TDW, indicating that most of the tested traits were significantly affected by drought. Medium to high heritability estimates were obtained for most of the traits, and heritability was higher for five traits under NC and seven traits under SC. Heritability ranged from 0.333 to 0.971 under NC and 0.331 to 0.983 under SC (Table 1). Pearson correlations were calculated among all traits, and we found 56 and 50 significant correlation coefficients (P < 0.05) under NC and SC, respectively (Table S1).

**Table 1:** Phenotypic variation in 13 traits in 373 *Aegilops tauschii* accessions under the normal condition (NC) and the PEG-induced, simulated drought-stress condition (SC).

Trait	Condition	Mean ± s.d.	CV(%)	F-value	*h*_*B*_(%)^a^
RDW	NC	0.016 ± 0.009	55.983	48.191**	0.431
	SC	0.013 ± 0.009	70.672		0.440
SDW	NC	0.041 ± 0.020	49.342	21.498**	0.552
	SC	0.022 ± 0.011	49.682		0.552
DRS	NC	0.419 ± 0.285	67.962	37.497**	0.719
	SC	0.987 ± 1.792	181.476		0.822
RFW	NC	0.276 ± 0.130	47.209	0.287^ns^	0.964
	SC	0.108 ± 0.048	43.921		0.958
SFW	NC	0.278 ± 0.145	52.219	1.335**	0.924
	SC	0.073 ± 0.034	46.294		0.920
FRS	NC	1.073 ± 0.649	60.544	0.142^ns^	0.971
	SC	1.572 ± 0.556	35.415		0.983
SH	NC	17.267 ± 3.998	23.155	6.833**	0.333
	SC	13.785 ± 3.196	23.185		0.337
RL	NC	246.692 ± 129.523	52.504	20.049**	0.341
	SC	340.228 ± 415.846	122.226		0.331
RD	NC	7.749 ± 33.842	436.727	10.66**	0.475
	SC	3.481 ± 10.981	315.422		0.440
TDW	NC	0.057 ± 0.025	44.074	1.521^ns^	0.862
	SC	0.035 ± 0.014	39.802		0.902
TFW	NC	0.554 ± 0.264	47.622	0.592^ns^	0.666
	SC	0.182 ± 0.075	41.300		0.927
RT	NC	1229.254 ± 912.330	74.218	58.931**	0.343
	SC	2180.079 ± 3181.680	145.943		0.334
TNOR	NC	2148.141 ± 864.048	74.578	58.574**	0.342
	SC	1158.575 ± 3163.958	147.288		0.355

RFW: root fresh weight; SFW: shoot fresh weight; FRS: root to shoot ratio of fresh weight; RDW: root dry weight; SFW: shoot dry weight; FRS: root to shoot ratio of dry weight; SH: shoot height; TFW: total fresh weight; TDW: total dry weight; TRL: total root length; RD: root diameter; RT: number of root tips; TNOR: the number of root in diameter 0.000 to 0.500.

a Broad-sense heritability of the tested traits.

** significant at *p* < 0.01;

ns: not significant.

### Principal component analysis (PCA) and multiple linear stepwise regressions

PCA were performed for all traits using DI (Table 2) that were highly correlated according to the Bartlett's test of sphericity (χ^2^ = 5056.738; P < 0.001). To establish selection indices involving multiple drought-resistance traits, a series of linear regressions were performed for all traits. We built the regression to explain TDW and chose our predictive variables through stepwise regression (Table 3). The final stepwise model explained 93.9% and 65.3% of the phenotypic variation in TDW under NC and SC, respectively. The model contained nine traits for NC (RFW, RDW, FRS, DRS, TFW, RD, RL, RT, and TNOR) and seven traits for SC (RFW, RDW, FRS, DRS, TFW, RL, and TNOR).

**Table 2:** Principal component analysis (PCA). For trait abbreviations see Table 1.

	Trait	PC 1	PC 2	PC 3	PC 4
	RFW	0.655	-0.082	0.618	0.238
	SFW	0.584	-0.179	-0.144	-0.264
	FRS	-0.050	0.084	0.831	0.469
	RDW	0.734	-0.348	-0.210	0.350
	SDW	0.365	0.244	0.365	-0.677
	DRS	0.483	-0.411	-0.400	0.495
Characteristic vector	SH	0.608	-0.042	-0.132	-0.282
	TFW	0.865	-0.166	0.086	0.024
	TDW	0.815	-0.014	0.094	-0.265
	RL	0.278	0.765	-0.111	0.173
	RD	0.083	-0.362	-0.065	-0.005
	RT	0.294	0.891	-0.170	0.157
	TNOR	0.295	0.891	-0.167	0.154
Eigenvalues		3.720	2.731	1.538	1.400
Contribution %		28.614	21.005	11.831	10.766
Cumulative contribution %		28.614	49.618	61.449	72.215

**Table 3:** Multiple linear stepwise regression to explain total dry weight (TDW) from root traits built with *Aegilops tauschii* genotypes means. For trait abbreviations see Table 1.

Treatment	Final stepwise model	R^2^	P value
NC	TDW = 0.011 – 0.08RFW + 2.014RDW + 0.02FRS – 0.032DRS + 0.089TFW + 0.00005817RD – 0.000002274RL -0.000001614RT + 0.000008294TNOR	0.939	< 0.001
SC	TDW = 0.011 – 0.033RFW + 0.92RDW – 0.001FRS – 0.003DRS – 0.105TFW + 0.000002321RL + 0.000002292TNOR	0.653	< 0.001

We performed a comprehensive evaluation of drought resistance in *A. tauschii* using D values and DI ( Table S2). Among the 373 *A. tauschii* accessions, AS623213 that had the highest D value and AS623095 that had the lowest D value were selected as extremely resistant and susceptible genotypes, respectively. Overall, we identified six genotypes (1.6%) with high resistance (D ≥ 0.5), 262 (70.2%) with moderate resistance (0.30 ≤ D < 0.5), and 105 (28.2%) with low resistance (D < 0.30). Next, we observed that *A. tauschii* accessions with a higher D value also had a higher DI (Table S2), which suggested that the two selection indicators were effective for screening *A. tauschii* under SC.

### Marker-trait association analysis

The Bonferroni-corrected threshold (-log *p* > 3.839, *α* = 1) was used as the cutoff value for identifying marker-trait associations (Yang *et al.*, 2014). A total of 208 and 28 SNPs were detected by the GLM and MLM, respectively, while 25 significant SNPs with genome-wide distribution (chromosomes [Chr.] 1D-7D) markers were detected by both models (Table 4; Figure S1 and Table S3).

**Table 4:** Genome-wide association of 13 tested traits under the normal condition (NC) and the PEG-induced, simulated drought-stress condition (SC) detected using general linear (GLM) and mixed linear (MLM) models. For trait abbreviations see Table 1.

	Trait	GLM					MLM					No. Share^c^
		No.sig^a^	Average -log(*P*)	Range -log(*P*)	Average R^2^ (%)^b^	Range R^2^ (%) b	No.sig^a^	Average -log(*P*)	Range -log(*P*)	Average R^2^ (%) b	Range R^2^ (%)^b^	
**NC**	FRS	31	4.476	3.843-5.522	4.958	4.183-6.240	1	3.970		4.732		1
	RD	9	4.055	3.884-4.334	4.367	4.160-4.702						
	RDW	1	4.314		4.891							
	RFW	28	4.555	3.873-6.217	5.087	4.243-7.128						
	RL	16	4.734	3.866-7.607	4.912	3.896-8.144						
	RT	12	4.635	3.858-5.551	4.674	3.866-6.016	1	3.980		4.805		
	SDW	5	4.703	3.855-6.332	4.983	3.983-6.860	1	4.040		4.803		1
	SFW	7	4.564	3.878-6.596	4.883	4.074-7.277	2	4.122	4.109-4.136	4.932	4.912-4.951	2
	SH	1	3.932		4.410							
	TDW	9	4.567	3.901-6.883	4.826	4.044-7.508	1	4.217		5.033		1
	TFW	21	4.763	3.875-6.930	5.116	4.062-7.653	2	3.893	3.857-3.930	4.566	4.516-4.616	2
	TNOR	11	4.701	3.873-5.462	4.728	3.780-5.896	1	3.945		4.760		1
**SC**	DRS						1	4.238		7.197		
	FRS	1	4.242		4.588							
	RD	8	5.628	3.875-7.932	6.569	4.319-9.367	6	5.793	3.844-6.505	8.140	4.995-9.211	5
	RDW	6	4.184	3.959-5.076	4.404	4.129-5.395						
	RT	1	3.967		4.460							
	SFW	1	3.991		4.339							
	TDW	8	4.561	4.006-5.631	4.898	4.238-6.162	2	4.087	3.992-4.183	4.857	4.725-4.989	2
	TFW	6	4.447	3.868-5.290	4.792	4.112-5.796	3	4.678	4.089-4.973	5.637	4.813-6.049	3
	TNOR	1	4.148		4.708							
**DI**	DRS	1	4.639		5.288		1	5.286		9.930		1
	FRS	7	4.264	3.868-5.330	4.965	4.370-6.229						
	RD	3	4.432	4.432-4.432	5.154	5.154-5.154	3	4.225	4.225-4.225	5.133	5.133-5.133	3
	RL	1	4.425		4.979		1	3.848		4.513		1
	RT	3	4.323	3.872-4.906	5.228	4.415-6.447	1	4.401		5.838		1
	SDW	5	4.850	4.421-5.085	5.625	5.064-5.907						
	TDW	2	4.274	4.059-4.490	4.902	4.604-5.199						
	TNOR	3	4.366	3.982-4.872	5.280	4.554-6.395	1	4.396		5.818		1
**Total**		**208**					**28**					**25**

a Total number of significantly associated SNPs detected by GLM and MLM at the threshold of -log^10^ *p* = 3.839

b R^2^ value showing the percentage of explained phenotypic variation

c Number of significant SNPs detected by both models

**Figure 1:**
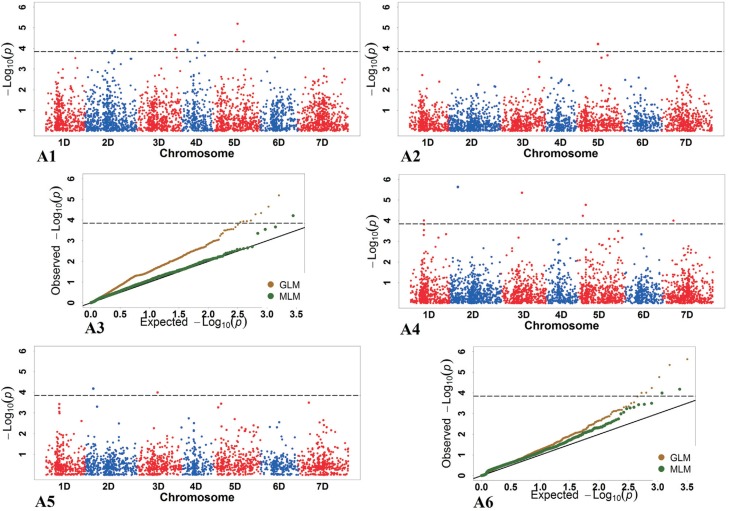
The p values of the SNPs and quantile-quantile (Q-Q) plots of p values for total dry weight (TDW) under the normal condition (NC) and the PEG-induced, simulated drought-stress condition (SC). Summary of GWAS results for TDW. A1 and A2) GLM and MLM results for association under NC condition. A3) Q-Q plots of GLM and MLM under NC condition. A4 and A5) GLM and MLM results for association under SC condition. A6) Q-Q plots of GLM and MLM under SC condition.

Under NC, significant markers were detected by both the GLM and MLM for FRS, RT, SDW, SFW, TDW, TFW, and TNOR (Table 4), and by the GLM for RD, RDW, RFW, RL, and SH (partly shown in Figure 1). No significant markers were detected for FRS by any of the two models.

Under SC, significant markers were detected by both the GLM and MLM for RD, TDW, and TFW, and by the GLM for FRS, RDW, RT, SFW, and TNOR (partly shown in Figure 1). No significant markers were detected for RFW, RT, SH, and SDW by any of the two models.

Numerous SNPs were significantly associated with the DI in both the GLM and MLM, and a relatively large amount of phenotypic variation in DI was explained by the studied markers (Table 4).

We performed a BLAST search against the IWGSC using the SNP sequences, and we found that their chromosomal locations were different from those of the best hits returned from IWGSC. For example, the SNP markers *contig10767_892* and *contig50332_70* located on Chr. 7D and 6D, respectively, on the genetic map of Luo *et al.* (2014) were located on Chr. 5DL and 6BL, respectively, according to the IWGSC BLAST results.

### QTLs and putative candidate genes associated with significant loci

To compare the identified regions between the 373 *A. tauschii* accessions, markers separated by less than 5 cM were considered to be part of the same QTL (Massman *et al.*, 2011). The results revealed three QTLs that were related to RD-SC, RD-DI, and TFW-SC. To find candidate genes, flanking genes, and trait-related proteins, we performed a BLAST search of the NCBI database using the IWGSC BLAST results and then a direct BLASTX search of the NCBI database. Putative and flanking genes associated with significant loci are listed in Table S3. We identified several candidate genes that were associated with different traits. Examples include *Rht-A* that was associated with TFW-SC, RD-SC, TNOR-NC, SDW-NC, SFW-NC, TDW-NC, and TFW-NC; *Rht-B* associated with TFW-SC; *Glo-2* associated with TFW-SC and TDW-NC; *WM1.7* associated with RD-SC and RD-DI; and *Acc-2* associated with RD-SC, RD-DI, TDW-SC, TNOR-NC, and FRS-DI. We also found two candidate vernalization-requirement genes, *VRN2* and *VRN-B1*, suggesting that vernalization might be related to drought resistance.

We also identified a few putative candidate genes associated with phenotypic traits. These genes could be roughly divided into three groups: the first group included genes encoding enzymes, such as *RUBISCO*, *CKX2.5*, *Acc-1* and *Acc-2*, suggesting that many biochemical pathways were activated under SC; the second group included genes encoding storage proteins, such as *Glo-2*, *WM1.12*, and *WM1.7*, which might be activated in response to drought stress; and the final group included genes encoding drought-induced proteins, such as *Hotr1*, *Rht-A*, *Rht-B*, *VRN-B1*, and *VRN2*, that might play a crucial role in the drought-resistance reaction of *A. tauschii*.

## Discussion

### Importance of the wheat wild relative *A. tauschii*

*A. tauschii* possesses numerous traits of high agronomic interest, such as yield, insect resistance, disease resistance, and drought resistance (Cox, 1994; Ma *et al.*, 1995; Assefa, 2000; Aghaee-Sarbarzeh *et al.*, 2002), and its genes can be incorporated into the wheat genome via intergenic crossing (Valkoun *et al.*, 1990; Cox *et al.*, 1992; Li *et al.*, 2006; Zhang and Ma, 2008). Many agronomically useful traits have been already incorporated into wheat (Raupp *et al.*, 1993; Cox and Hatchett, 1994; Friebe *et al.*, 1996). In addition, artificial hybridization between tetraploid wheat and *A. tauschii* has resulted in allohexaploid wheat lines, known as 'resynthesized' or 'synthetic hexaploid' wheat (SW) (Mujeeb-Kazi *et al.*, 1996), i.e. 'Chuanmai 42' (CM42), which is derived from a cross between *Triticum durum* and *A. tauschii* and is resistant to Chinese new stripe rust races (Li *et al.*, 2006).

Based on the results of this study, we believe that drought resistance is another *A. tauschii* trait that could be incorporated into the wheat breeding programs. We identified *A. tauschii* accessions with high drought resistance that could be used as germplasm resources to widen the genetic diversity of cultivated wheat and, thus, to reduce the time required to breed for drought resistance.

### Loci controlling drought resistance traits

Here, we reported the outcome of a genome-wide association study for the identification of genomic regions in *A. tauschii* responding to NC and SC. AM involved 7,185 SNP markers genotyped in a core collection of 373 *A. tauschii* accessions. Linkage mapping using different segregation populations tested in different environments could be also applied to detect QTLs, but there are only a few reports on QTL mapping related to drought-resistance traits in *A. tauschii*, compared with the high number of such studies in wheat using linkage mapping.

Landjeva *et al.* (2008) detected QTLs for RL on Chr. 1A, 6D, and 7D under SC, while Zhang *et al.* (2013) found two QTLs for RL associated with drought resistance on Chr. 6D in two F_8:9_ recombinant inbred line populations (Weimai 8 x Yannong 19 and Weimai 8 x Luohan 2). In our study, we also identified a significant locus (*contig03437_336*) on Chr. 6D (28.073 cM) that was associated with RL-DI, and we also found two loci related to RD-SC and RD-DI on Chr. 7D. However, Liu *et al.* (2013) found QTLs for RL on Chr. 2D and 5D under two different water conditions. Quarrie *et al.* (2005) mapped QTLs for drought resistance in hexaploid wheat on Chr. 2D and 3D, and found that three yield QTL clusters were coincident with *Vrn-A1* on Chr. 5AL and *Vrn-D1* on Chr. 5DL. By comparison, we identified seven significant loci on Chr. 2D and one significant locus on Chr. 2D. Furthermore, we found a candidate *VRN2* at the significant loci *GCE8AKX01BMYMJ_66* and *GDEEGVY01D8PT5_76* located on Chr. 5D and associated with RD-SC and RD-DI. These results indicated that vernalization-required genes probably affect drought resistance in wheat. These findings further suggested the importance of exploring the relationship between drought resistance and vernalization-required genes.

Significant genome-wide loci were detected by both the GLM and MLM. Some traits were associated with multiple chromosomes, including RD-DI associated with SNPs on Chr. 1D and 6D, TFW-NC associated with SNPs on Chr. 1D and 5D, and RD-NC associated with SNPs on Chr. 4D, 5D, and 7D. Massman *et al.* (2011) stated that significant SNP markers separated by less than 5 cM could be considered as a single QTL. Accordingly, GCE8AKX02IHJOC_389, *contig37658_165*, and GA8KES402HD74L_87 (Chr. 1D) separated by less than 1 cM were considered as a single QTL related to TFW-SC. Similarly, GCE8AKX01BMYMJ_66 and GDEEGVY01D8PT5_76 (Chr. 5D) also separated by less than 1 cM were considered as a single QTL related to RD-DI and RD-SC (Table S3).

Until the wheat genome map is complete, loci identified in this study as associated with drought resistance traits cannot be directly compared with QTLs reported by previous studies in wheat. In addition, since the genome of *A. tauschii* is not equivalent to the D-genome of wheat, only approximate chromosomal locations that control drought resistance traits can be inferred. For example, *contig10767_892* located on Chr. 7D in *A. tauschii* was found on Chr. 5DL in hexaploid wheat. Similarly, *contig50332_70* located on Chr. 6D in *A. tauschii* was found on Chr. 6BL in wheat. One possible reason for these differences could be the translocation of chromosomal regions during the hexaploidization of common wheat, in which *A. tauschii* was involved.

### Analysis of putative candidate and flanking genes

Drought resistance is a complex trait resulting from the interaction of root and shoot traits. In response to drought stress, wheat has developed highly specialized morphological, physiological and biochemical mechanisms to increase the efficiency of nutrient and water acquisition from soil (Ludlow and Muchow 1990; Richards *et al.*, 2002; Nicotra and Davidson, 2010). These mechanisms are closely associated with genes controlling drought resistance and apparently responsive traits under drought conditions. Previous studies have reported many genes related to drought resistance in wheat, such as *DREB* that plays a central role in plant stress response (Agarwal *et al.*, 2006; Mizoi *et al.*, 2012) and *TaAIDFa* that encodes a C-repeat/dehydration-responsive element-binding factor responsive to drought (Xu *et al.*, 2008). In addition, the silencing of *TaBTF3* impairs resistance to drought stress, suggesting that it may be involved in abiotic stress response in higher plants (Kang *et al.*, 2013). Jiang *et al.* (2014) isolated a strongly drought-induced C3H zinc finger gene, *AetTZF1*, in *A. tauschii*. Uga *et al.* (2013) characterized the *DRO1* gene that controls root growth angle in rice, which was the first root QTL that cloned in a crop species. Rice OsTZF1 confers increased stress resistance to drought by regulating stress-related genes (Jan *et al.*, 2013).

In this study, we identified several putative candidate genes associated with phenotypic traits related to drought resistance. These genes could be broadly divided into three groups: (1) genes related to various enzymes, suggesting that many biochemical pathways are activated under drought conditions; (2) genes related to storage proteins that may be synthesized in response to drought stress; and (3) genes related to drought-induced proteins that probably play a crucial role in drought resistance. These findings reflected the complexity of drought-resistance mechanisms and the large number of genes involved in these mechanisms. Information on SNPs and genes related to drought-resistance might provide a genetic basis for gene cloning and marker-assisted selection in the wheat breeding programs.

## Conclusion

We performed a genome-wide association study for drought resistance traits in a population of 373 *A. tauschii* accessions using 7,185 SNP markers and we detected 25 significant markers using GLM and MLM analysis. Furthermore, we identified candidate genes at significant loci and their flanking regions that might control drought resistance traits, including genes encoding enzymes, storage proteins, and drought-induced proteins. The results provided essential information on SNPs and genes related to drought resistance in *A. tauschii* that could be used for breeding drought-resistant wheat cultivars.

## Supplementary Material

The following online material is available for this article:

Table S1 - Genetic correlation among selected traits

Table S2 - Top 10 and bottommost 10 genotypes on DI and D value

Table S3 - Significant SNPs and candidate genes

Figure S1 - The p values of the SNPs and quantile-quantile (Q-Q) plots

This material is available as part of the online article from http://www.scielo.br/gmb
