# Supplementary material for: Genome-wide association study of drought-related resistance traits in Aegilops tauschii
Source: Genet Mol Biol. 2016 Jul 7;39(3):398–407. doi: 10.1590/1678-4685-GMB-2015-0232 (PMC5004832; doi:10.1590/1678-4685-GMB-2015-0232)
Supplement: Supplementary file 1 [file 1415-4757-gmb-1678-4685-GMB-2015-0232-Suppl01.pdf]

Table S1 - Genetic correlation among selected traits under the normal condition (NC, above the diagonal) and the PEG-induced, simulated drought-stress condition (SC, bellow the diagonal).

	RFW	SFW	FRS	RDW	SDW	DRS	SH	TFW	TDW	RL	RD	RT	TNOR
RFW	1	0.834**	0.035	0.466**	0.838**	–0.133**	0.569**	0.953**	0.408**	0.844**	–0.076	0.243**	0.249**
SFW	0.658**	1	–0.236**	0.343**	0.902**	–0.202**	0.664**	0.962**	0.449**	0.853**	–0.061	0.079	0.088
FRS	0.376**	–0.372**	1	0.038	–0.125*	0.096	–0.062	–0.113*	–0.093	–0.089	–0.022	0.115*	0.114*
RDW	0.448**	0.414**	0.002	1	0.385**	0.633**	0.321**	0.419**	0.252**	0.660**	–0.037	0.292**	0.290**
SDW	0.387**	0.489**	–0.102	–0.068	1	–0.304**	0.578**	0.910**	0.398**	0.947**	–0.054	0.122*	0.129*
DRS	0.148**	0.148**	–0.056	0.625**	–0.457**	1	–0.097	–0.177**	–0.013	–0.028	–0.013	0.079	0.074
SH	0.446**	0.600**	–0.166**	0.310**	0.309**	0.052	1	0.646**	0.269**	0.582**	0.012	0.345**	0.352**
TFW	0.716**	0.713**	0.011	0.430**	0.342**	0.138**	0.459**	1	0.448**	0.886**	–0.071	0.164**	0.171**
TDW	0.122*	0.01	0.195**	0.056	0.149**	–0.139**	0.075	0.096	1	0.411**	–0.210**	0.204**	0.209**
RL	0.496**	0.536**	–0.045	0.557**	0.550**	0.063	0.375**	0.694**	0.167**	1	–0.056	0.201**	0.206**
RD	0.127*	0.135**	–0.047	0.261**	–0.024	0.210**	0.081	0.143**	–0.176**	0.148**	1	0.017	0.015
RT	0.098	0.091	0.066	0.045	0.175**	–0.114*	0.084	0.109*	0.835**	0.173**	–0.126*	1	0.998**
TNOR	0.097	0.09	0.065	0.042	0.175**	–0.115*	0.083	0.107*	0.832**	0.172**	–0.127*	0.999**	1

RFW: root fresh weight; SFW: shoot fresh weight; FRS: root to shoot ratio of fresh weight; RDW: root dry weight; SFW: shoot dry weight; DRS: root to shoot ratio of dry weight; SH: shoot height; TFW: total fresh weight; TDW: total dry weight; RL: root length; RD: root diameter; RT: number of root tips; TNOR: the number of root in diameter 0.000 to 0.500; * and ** represent significance level of $P < 0.05$ and $P < 0.01$ respectively.
