# Supplementary material for: Genome-wide association study of drought-related resistance traits in Aegilops tauschii
Source: Genet Mol Biol. 2016 Jul 7;39(3):398–407. doi: 10.1590/1678-4685-GMB-2015-0232 (PMC5004832; doi:10.1590/1678-4685-GMB-2015-0232)
Supplement: Supplementary file 2 [file 1415-4757-gmb-1678-4685-GMB-2015-0232-Suppl02.pdf]

Table S2 - Top 10 genotypes with extremely high resistance and bottommost 10 genotypes with extremely low resistance selected from 373 *Aegilops tauschii* based on the drought resistance index (DI) and D value.

**Genotype**	DI													**D value**
	RFW	SFW	FRS	RDW	SDW	DRS	SH	TFW	TDW	RL	RD	RT	TNOR	
AS623213	0.439	0.518	0.847	0.957	1.180	0.810	1.302	0.476	1.119	45.438	0.023	29.662	30.707	0.674
AS623036	1.370	1.188	1.154	6.000	0.143	42.000	1.527	1.302	1.444	0.102	10.801	1.142	1.203	0.595
AS623146	0.294	0.352	0.835	0.351	6.481	0.054	0.816	0.316	3.989	4.301	0.125	7.603	8.223	0.558
AS623395	1.381	0.394	3.505	1.811	1.076	1.683	0.739	0.843	1.310	14.319	0.135	18.181	19.137	0.542
AS623024	1.414	1.247	1.134	12.000	0.333	36.000	1.191	1.333	2.667	0.186	2.152	0.941	0.904	0.522
AS623417	0.636	0.534	1.192	1.641	1.070	1.533	0.959	0.580	1.226	0.032	16.992	0.297	0.297	0.501
AS623035	2.256	1.591	1.155	5.890	0.618	9.851	1.084	1.910	2.320	0.849	0.262	0.589	0.621	0.498
AS623101	0.938	1.250	0.750	6.000	0.500	12.000	1.072	1.071	1.875	2.362	1.669	1.877	1.768	0.495
AS623212	0.290	0.282	1.027	0.667	0.641	1.040	0.934	0.286	0.647	14.807	0.025	20.563	20.928	0.486
AS623419	1.036	0.410	2.527	1.689	1.012	1.668	0.939	0.694	1.211	22.189	0.024	10.027	10.853	0.484
AS623245	0.177	0.097	1.826	0.744	0.347	2.142	0.421	0.164	0.388	0.411	0.848	0.330	0.321	0.268
AS623116	0.159	0.110	1.440	0.294	0.231	1.276	0.620	0.137	0.246	0.217	1.486	0.174	0.167	0.268
AS623167	0.271	0.147	1.841	0.364	0.309	1.176	0.528	0.206	0.321	0.259	0.349	0.497	0.529	0.268
AS623278	0.202	0.130	1.561	0.475	0.290	1.637	0.593	0.187	0.268	0.177	0.928	0.119	0.113	0.267
AS623246	0.217	0.090	2.398	0.203	0.422	0.480	0.532	0.105	0.331	0.484	0.750	0.333	0.319	0.267
AS623029	0.172	0.256	0.674	0.500	0.118	4.250	0.551	0.208	0.240	0.299	0.823	0.413	0.440	0.266
AS623065	0.207	0.091	2.267	0.444	0.333	1.333	0.557	0.152	0.364	0.073	0.975	0.029	0.026	0.266
AS623407	0.167	0.304	0.550	0.261	0.046	5.699	0.708	0.242	0.109	0.667	0.510	0.197	0.193	0.265
AS623243	0.106	0.056	1.912	0.667	0.207	3.228	0.482	0.092	0.282	0.554	0.806	0.670	0.651	0.264
AS623095	0.146	0.146	1.001	0.444	0.116	3.833	0.527	0.146	0.229	0.572	0.073	0.839	0.929	0.260

RFW: root fresh weight; SFW: shoot fresh weight; FRS: root to shoot ratio of fresh weight; RDW: root dry weight; SFW: shoot dry weight; DRS: root to shoot ratio of dry weight; SH: shoot height; TFW: total fresh weight; TDW: total dry weight; RL: root length; RD: root diameter; RT: number of root tips; TNOR: the number of root in diameter 0.000 to 0.500.
