# Supplementary figures and images for: Genome-wide association study of drought-related resistance traits in Aegilops tauschii
Source: Genet Mol Biol. 2016 Jul 7;39(3):398–407. doi: 10.1590/1678-4685-GMB-2015-0232 (PMC5004832; doi:10.1590/1678-4685-GMB-2015-0232)

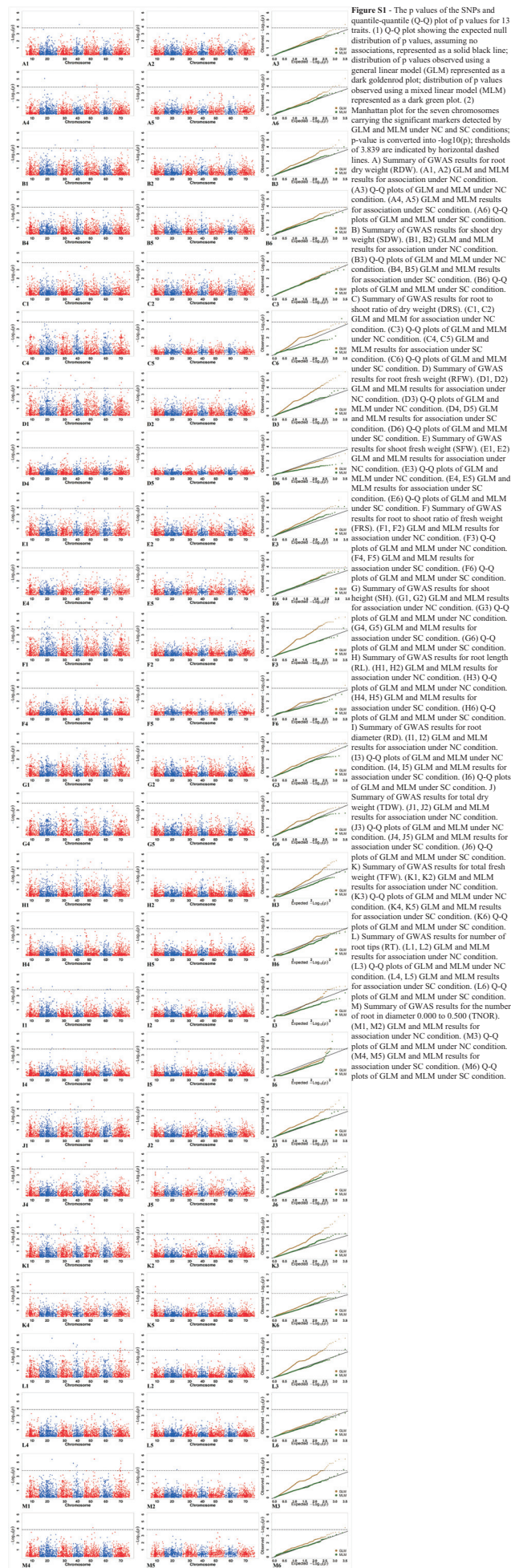

Supplement: Supplementary file 3 [file 1415-4757-gmb-1678-4685-GMB-2015-0232-Suppl04.pdf]
